# A coma-free super-high resolution optical spectrometer using 44 high dispersion sub-gratings

**DOI:** 10.1038/s41598-020-80307-z

**Published:** 2021-01-13

**Authors:** Hua-Tian Tu, An-Qing Jiang, Jian-Ke Chen, Wei-Jie Lu, Kai-Yan Zang, Hao-Qi Tang, Osamu Yoshie, Xiao-Dong Xiang, Young-Pak Lee, Hai-Bin Zhao, Yu-Xiang Zheng, Song-You Wang, Junpeng Guo, Rong-Jun Zhang, Jing Li, Yue-Mei Yang, W. D. Lynch, Liang-Yao Chen

**Affiliations:** 1 Department of Optical Science and Engineering, Fudan University, Shanghai, China; 2 Graduate School of IPS, Waseda University, Fukuoka, Japan; 3 Department of Material Science and Engineering, SUSTC, Shenzhen, China; 4 Department of Physics, Hanyang University, Seoul, Korea; 5 Department of Electrical and Computer Engineering, University of Alabama in Huntsville, Huntsville, AL 35899 USA; 6 Department of Physics, Iowa State University, Ames, IA USA

**Keywords:** Spectrophotometry, Spectrophotometry, Imaging and sensing

## Abstract

Unlike the single grating Czerny–Turner configuration spectrometers, a super-high spectral resolution optical spectrometer with zero coma aberration is first experimentally demonstrated by using a compound integrated diffraction grating module consisting of 44 high dispersion sub-gratings and a two-dimensional backside-illuminated charge-coupled device array photodetector. The demonstrated super-high resolution spectrometer gives 0.005 nm (5 pm) spectral resolution in ultra-violet range and 0.01 nm spectral resolution in the visible range, as well as a uniform efficiency of diffraction in a broad 200 nm to 1000 nm wavelength region. Our new zero-off-axis spectrometer configuration has the unique merit that enables it to be used for a wide range of spectral sensing and measurement applications.

## Introduction

**Figure 1:**
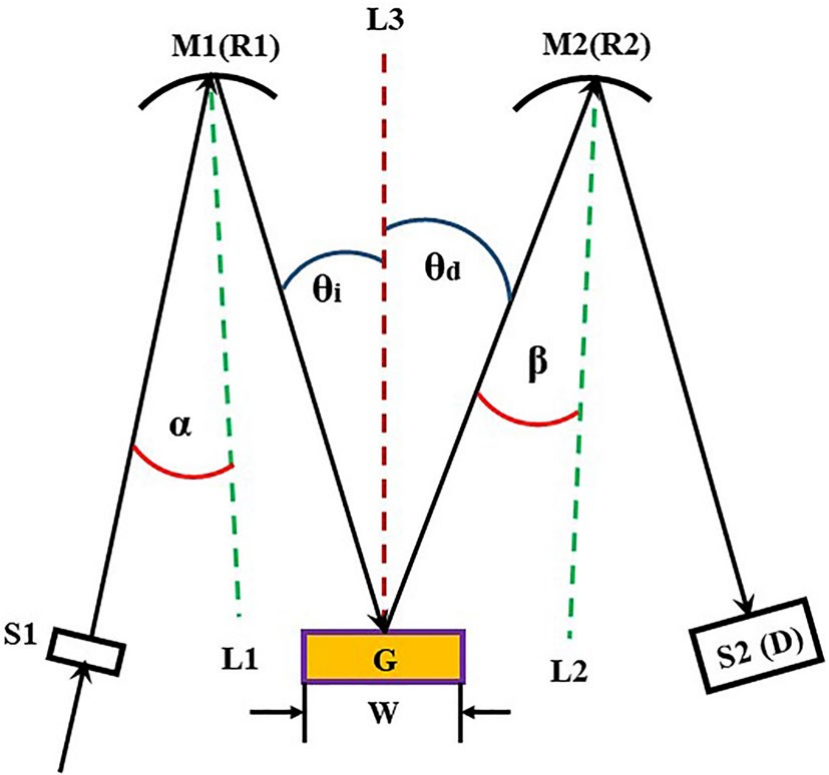
A typical configuration of a Czerny-Turner spectrometer, where G is the plane grating with a width of w; M1 and M2 are two spherical mirrors with radiuses of *R*_1_ and *R*_2_, respectively; S1 is the entering slit of the light beam; and S2 (or D) is the exit slit (or photon detector) located at the focal plane of the diffracted light reflected by M2. With respect to lines L1 and L2, which are normal to the surfaces of M1 and M2, respectively, the entering and diffracted light beams are obliquely incident to M1 and M2 at off-angles of *α* and *β* from the homocentric axis, respectively. With respect to line L3, which is normal to the grating surface, *θ*_*i*_ and *θ*_*d*_ are the incidence and diffraction angles of the light beams onto and out of the grating, respectively.

Super-high resolution optical spectrometers are significantly important for a wide range of applications in biochemical analysis, remote sensing, astronomy, and etc.^1^. Since the beginning of last century, many studies had been reported trying to solve the issue of astigmatic aberrations that occur as a result of reflection or refraction at a spherical surface, shifting the incident light beam obliquely off of the homocentric axis^2^. In optical systems, especially in high-precision optical instruments, astigmatic aberrations affect seriously the performance of the system. Moreover, these problems, which induce substantial measurement errors in a wide spectral range due to the strongly correlated restriction of the optical element arrangement in the system design, are difficult to solve. To date, the typical Czerny–Turner (C–T) configuration^3^, which consists of two spherical mirrors and a plane grating as shown in Fig. 1, is the most common configuration for spectroscopic analyses^1^. In the C–T configuration, the astigmatic aberrations, called the coma effect is an intrinsic issue that makes the spectral image out of focus at the focal plane of the diffracted light reflected from the mirror^1,3–18^.

For the Czerny-Turner configuration shown in Fig. 1, the coma width in the image Δ along the diffraction direction x can be evaluated as follows^8,10^:

1 $$\Delta = \frac{{3w^{2} R_{2} \cos^{2} \theta_{i} \cos \beta }}{{8\cos^{3} \alpha }}\left( {\frac{\sin \beta }{{R_{2}^{2} }} \cdot \frac{{\cos^{2} \theta_{d} \cos^{3} \alpha }}{{\cos^{2} \theta_{i} \cos^{3} \beta }} - \frac{{\sin \alpha \cos \theta_{i} }}{{R_{1}^{2} \cos \theta_{d} }}} \right).$$

Hereafter, two main approaches are studied to reduce the coma effect. (1) The coma effect can be reduced by decreasing the grating width *w*, since Δ is proportional to *w*^2^; however, using a low numerical aperture and compensation limits the spectral resolution, which is usually less than or equal to 0.1 nm in applications^1,3,16^. (2) The coma effect can be avoided by satisfying the condition Δ = 0, which reduces Eq. (1) to the following form^8,10^:

2 $$\frac{\sin \beta }{{\cos \alpha }} = \frac{{R_{2}^{2} \cos^{3} \beta \cos^{3} \theta_{i} }}{{R_{1}^{2} \cos^{3} \alpha \cos^{3} \theta_{d} }},$$

where the spectrometer parameters *α*, *β*, *R*_*1*_ and *R*_*2*_ can be optimized and fixed. However, the incidence angle *θ*_*i*_ and diffraction angle *θ*_*d*_ with respect to the axis normal to the plane of the grating must nonlinearly scan the entire spectral region with the variable values depending on the individual groove density *g* of the grating and the wavelength *λ* to perform the measurement on a variety of objectives, as shown by Eq. (3) ^13^.

3 $$\sin \theta_{i} - \sin \theta_{d} = m \cdot g \cdot \lambda ,$$

where *m* is the order of the grating diffraction and *g* is specified by the density of grooves of the grating (lines per mm). Therefore, according to Eqs. (2) and (3), the coma effect can be conditionally removed only at some specifically designed wavelength positions restricted within a relatively narrow spectral region.

Continuous researches are being made to make the feasibility and precision of spectrometers be improved in many different configurations and applications^19–32^. To exploit the usefulness and simplicity of a spectrometer in the C–T configuration while suppressing the corresponding intrinsic weaknesses arising from the coma effect, we propose a new spectrometer in an attempt to completely eliminate the root cause of the coma effect by making the off-axis angles *α* and *β* equal to 0, i.e., setting *α* = *β* = 0 in Eq. (1).

A two-dimensional (2D) advanced charge-coupled device (CCD) array detector was used in the entire 200–1000 nm wavelength region with a 2048 × 2048 pixel density and 13.5 × 13.5 μm^2^ pixel size^33^. A compound integrated module of the grating composed by 44 sub-gratings was designed and fulfilled to produce a spectrometer capable of identifying atoms based on their unique spectral “fingerprints”^19^ with ultrahigh resolution. Diffraction efficiency is relatively uniform over a broad wavelength region. Individual sub-gratings with high efficiency (order of *m* = 1) are used to make the spectra be multiply folded and imaged simultaneously on the focal plane of the photon detector. These sub-gratings can be chosen and arranged optimally at the blazed-wavelength *λ*_b_ according to the requirement of the system design without any moving part^23,34,35^.

In tests with a dual-element Hg-Ar lamp (Hg-Ar lamp (model HG-1), https://www.oceaninsight.com/products/light-sources/), the spectrometer shows the spectral data density evaluated by the highest k number, which is about 2.1 × 10^4^, 2.9 × 10^4^, and 2.7 × 10^4^, in the 200–305 nm, 305–593 nm and 593–1000 nm wavelength regions, respectively. Note that k = (total wavelength range)/(pixel resolution), which corresponds to a one-dimensional total of approximately 7.7 × 10^4^ wavelength data points measured once in the entire 200–1000 nm spectral region.

## Experimental results

**Figure 2:**
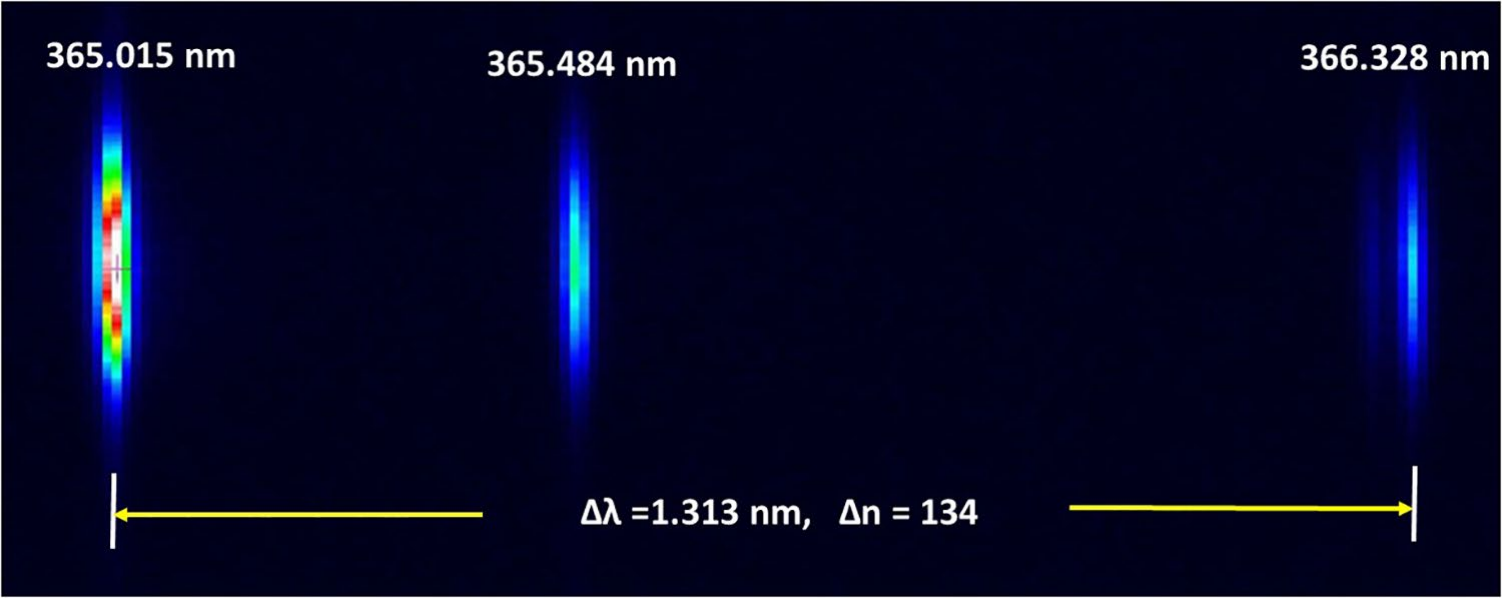
The image of the triple spectral lines of the Hg atom, obtained with ultrahigh resolution by CCD camera with elimination of the coma aberration in the zero-off-axis C–T configuration. These line structures occurred at 365.015 nm, 365.484 nm and 366.328 nm wavelengths, respectively, arise from excited emissions of the Hg atom and are intrinsically related to the electron transitions from the 6*d*_3_*D*_3_, 6*d*_3_*D*_2_ and 6*d*_1_*D*_2_ states to the 6*p*_3_*P*_2_ state. A very weak satellite line is clearly located at 366.288 nm, approximately 0.04 nm apart from the line at 366.328 nm.

By using a dual-element Hg–Ar lamp to test the astigmatic aberration of the spectrometer, the coma aberration has been successfully eliminated in the system with an integrated module of the grating composed by 44 sub-gratings in the zero-off-axis C–T configuration, as shown in Fig. 2. This figure presents typical images of the triple lines of the Hg atom at ultrahigh resolution. These line structures occurred at 365.015 nm, 365.484 nm and 366.328 nm wavelengths, respectively, arise from excited emissions of the Hg atom and intrinsically are related to the electron transitions from the 6*d*_3_*D*_3_, 6*d*_3_*D*_2_ and 6*d*_1_*D*_2_ states to the 6*p*_3_*P*_2_ state^36^. It can be recognized that there is a very weak satellite line located at 366.288 nm, approximately 0.04 nm apart from the line at 366.328 nm^19^. The number of pixels Δn between the lines at 365.015 nm and 366.328 nm wavelengths is 134, giving the dispersion of Δ*λ*/Δn = 0.0098 nm/pixel, which is in agreement with the designed value of the system.

**Figure 3:**
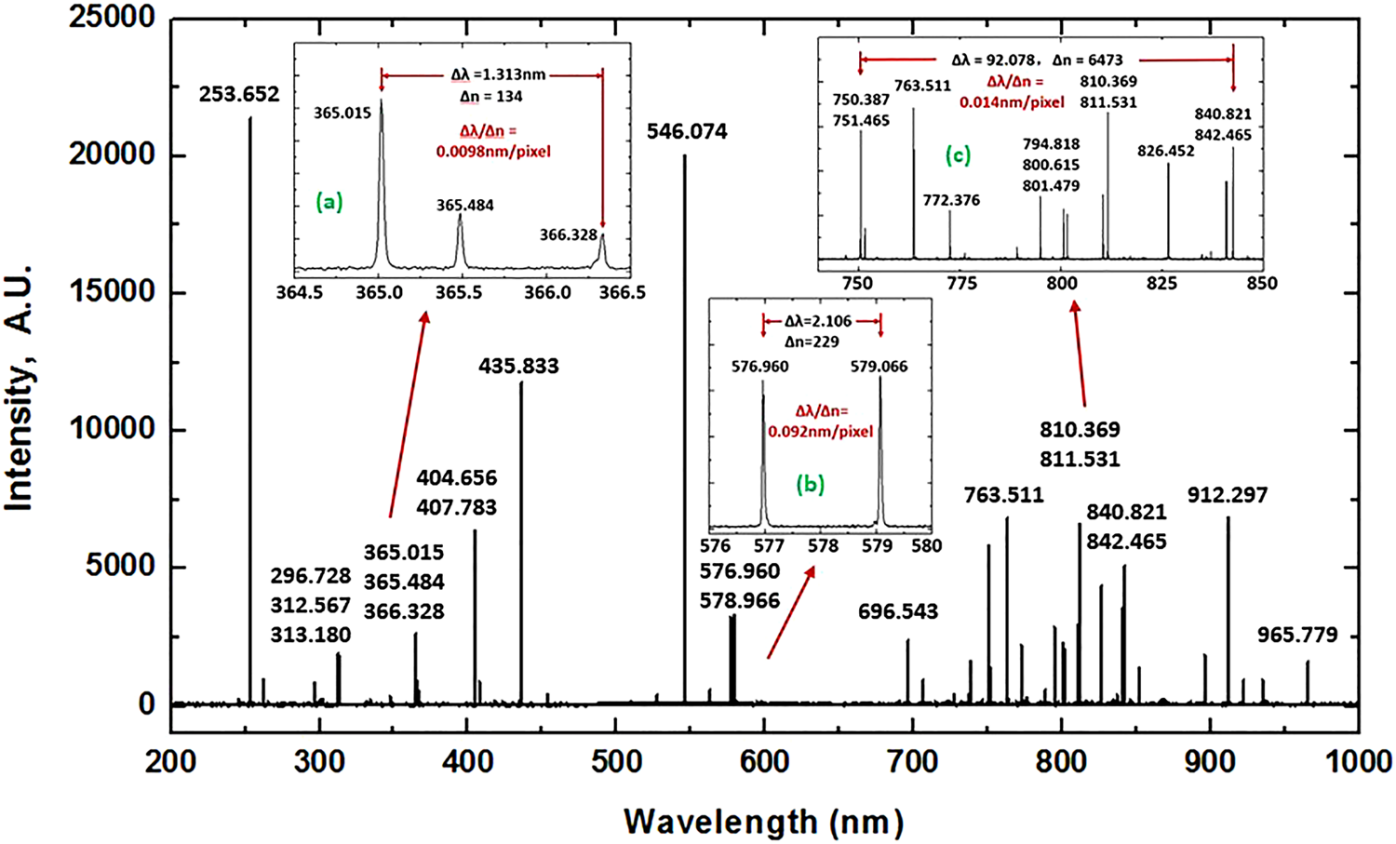
The spectrum of dual-element Hg–Ar light source which was applied to measure the wavelength lines in the 200–1000 nm spectral region, wherein the insets show magnified views of the well-resolved triple and twin spectral lines of elemental Hg and other spectral lines of elemental Ar.

The software for quick spectrum acquisition was designed, including the procedures of data measurement and calibration. To test the performance of the spectrometer, the Hg–Ar lamp with wavelength “fingerprints” of Hg and Ar atoms in the 200–1000 nm spectral range was applied. The detailed results are presented in Fig. 3, in which insets (a) and (b) give magnified structures of the featured triple and twin (corresponding to the transitions from the 6d_3_D_2_ and 6d_1_D_2_ states to the 6p_1_P_1_ state, respectively^36^) wavelength lines of elemental Hg and inset (c) shows magnified views of the elemental Ar lines in the 740–850 nm wavelength range.

The characteristic wavelength lines of the Hg and Ar atoms were evaluated to show the spectral resolution of the system. Figure 3 shows that these triple and twin line structures and the other atomic line structures can be finely recognized without ambiguity and are in consistency with the ones in expectation. By virtue of the implemented design considerations, the main feature line of 253.652 nm in the ultraviolet range for the Hg atom shows a higher strength due to the sub-gratings that were optimally arranged in the spectral positions.

**Figure 4:**
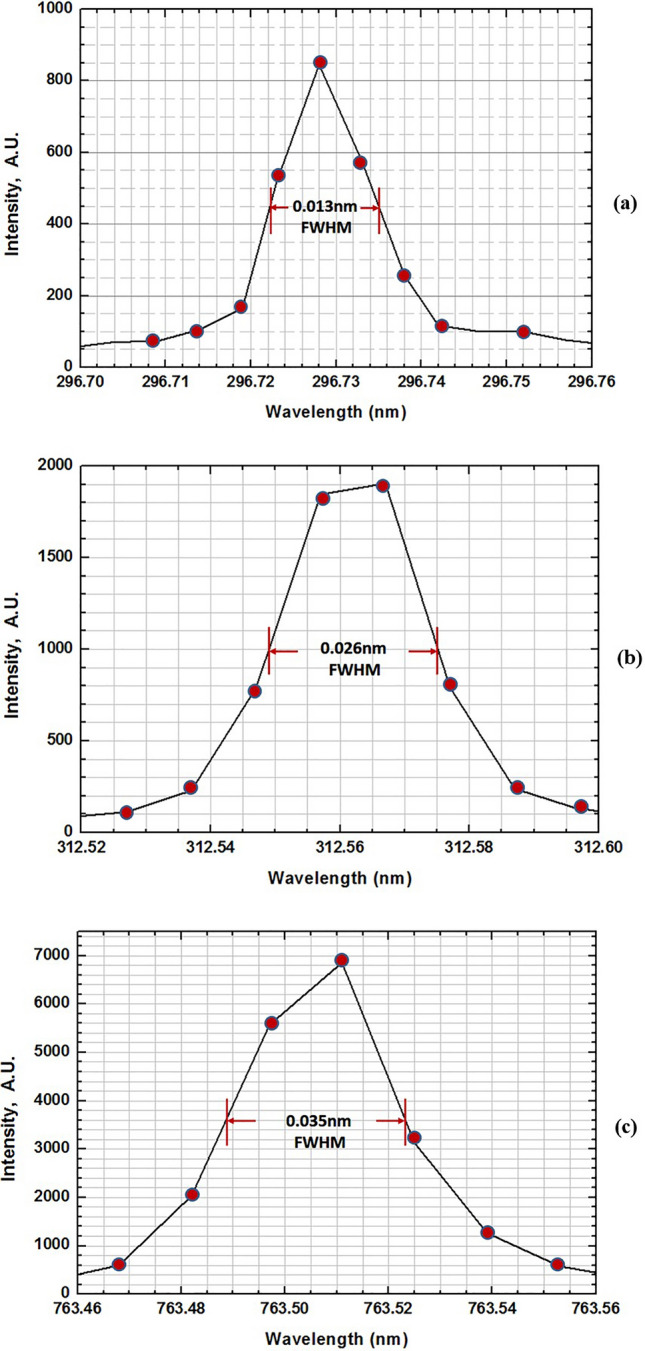
The spectra of fine wavelength lines of 296.728 nm and 312.567 nm for Hg atom, and as well as 763.511 nm for Ar atom were applied to test the resolution of the system, showing that the resolution per pixel (∆λ(FWHM) = 3δλ ≤ 3Δλ_max_) is better than 0.005 nm/pixel, 0.010 nm/pixel and 0.015 nm/pixel in the 200–300 nm (*g* = 3600 lines/mm), 300–600 nm (*g* = 1800 lines/mm) and 600–1000 nm (*g* = 1200 lines/mm) wavelength regions, respectively, which is consistent with the design expectations.

The wavelength resolution ∆*λ* is measured in experiment by the full width at half maximum (FWHM) of the wavelength line structure, and will be not equal to the resolution per pixel acting as the individual output slit located at the 2D focal image plane of the detector. Some weak satellite lines arise from multiple isotopes occurred in the Hg and Ar atoms and will make lines be broadened, resulting in error of measurement. As shown in Fig. 3, thus, lines of the structures occurred at 296.728 nm, 312.567 nm and 763.511 nm of Hg and Ar elements with the relatively fine and clean features were applied, respectively, to evaluate the spectral resolution with the results shown in Fig. 4. It at least needs 3 pixels, corresponding to 3 fixed slits of beam-exit, to cover the ∆*λ*(FWHM) of a spectral line. In terms of the spectral analysis, the values of ∆*λ*(FWHM) measured at 296.728 nm, 312.567 nm and 763.511 nm were determined to be approximately 0.013 nm, 0.026 nm and 0.035 nm, respectively. Hence, the resolution per pixel (∆*λ*(FWHM) = 3δ*λ* ≤ 3Δ*λ*_max_) will be higher than 0.005 nm/pixel, 0.010 nm/pixel and 0.015 nm/pixel in the 200–305 nm (*g* = 3600 lines/mm), 305–593 nm (*g* = 1800 lines/mm) and 593–1000 nm (*g* = 1200 lines/mm) wavelength regions, respectively, which is consistent with the design expectations.

## Discussions

**Figure 5:**
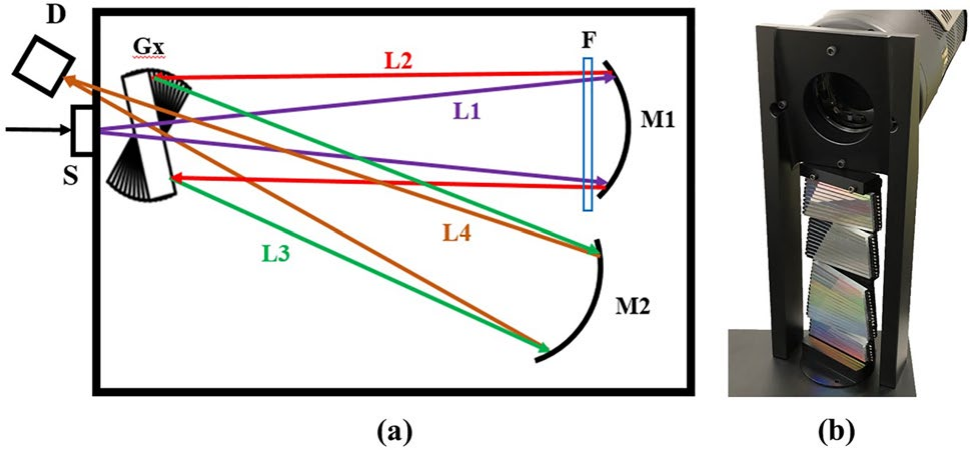
Schematic illustration of the high-resolution coma-free spectrometer. (**a**) The system configuration. S indicates a light beam slit, and Gx is the integrated module composed by 44 sub-gratings. The incident light enters along path L1 through a hole centered between gratings 22 and 23. The light along path L2 becomes parallel after reflected by spherical mirror M1 and then enters module Gx with an off-axis angle of *α* = 0. The grating-diffracted light along path L3 is then focused at the 2D focal plane of the CCD detector along path L4 after reflected by toroidal mirror M2 with an off-axis angle of *β* = 0. F is the set of filters applied to cut off the high order (*m* ≥ 2) of the diffracted light effectively in each sub-spectral range. (**b**) The camera with the 2D BSI-CCD array detector was placed on top of the grating module with a small off-axis tilt of approximately 6° in the direction perpendicular to the diffraction plane without the coma effect [The image of (**b**) was taken by the camera of iPhone-x with the background removed by the software of Pro_KnockOut_v3.2 (www.godimage.mobi)].

Arising from side effects, the measurement efficiency in optics in the ultraviolet and infrared regions usually will be poorer than that in the visible range. The intensity of light close to the center of the beam will be higher than the intensity that at the position of edge since the intensity of light is distributed non-uniformly in the fiber exit core area. In the configuration and design of the system, thus, the sub-gratings assembled in the integrated grating module Gx can be arranged flexibly. Those sub-gratings working in shorter (200–305 nm) and longer (593–1000 nm) wavelength ranges are put in the positions closer to the center of the light beam, as shown in Fig. 5 and Table 1. Each sub-grating is 70 mm long in the directions parallel to the incident plane of light, and is 2.9 mm high in the directions perpendicular to the incident plane of light (Optometrics, https://www.optometrics.com).

**Table 1:** Configuration of the grating module Gx with the dispersion features in each sub-spectral region for all 44 gratings arranged in the 200–1000 wavelength region.

1. Grating No	2. Start wavelength (λ_1_, nm)	3. End wavelength (λ_2_, nm)	4. Center wavelength (λ_o_, nm)	5. Spectral dispersion (nm/pixel)	6. Grating angle (θ_λ_, °)	7. Designed wavelength range (nm)	8. Redundant wavelength range (nm)
1	469.4	487.3	478.35	0.00950	25.500	17.9	19.46
2	451.4	469.4	460.4	0.00955	24.479	18	19.56
3	433.3	451.4	442.35	0.00959	23.460	18.1	19.65
4	415.2	433.3	424.25	0.00963	22.447	18.1	19.73
5	397	415.2	406.1	0.00967	21.438	18.2	19.81
6	378.8	397	387.9	0.00971	20.433	18.2	19.88
7	360.5	378.8	369.65	0.00974	19.432	18.3	19.94
8	342.2	360.5	351.35	0.00977	18.434	18.3	20.00
9	323.8	342.2	333	0.00979	17.439	18.4	20.05
10	305.4	323.8	314.6	0.00981	16.448	18.4	20.10
11	296.9	305.4	301.15	0.00453	32.824	8.5	9.29
12	288.3	296.9	292.6	0.00457	31.781	8.6	9.36
13	279.7	288.3	284	0.00460	30.744	8.6	9.43
14	271	279.7	275.35	0.00464	29.711	8.7	9.49
15	262.3	271	266.65	0.00467	28.683	8.7	9.56
16	253.5	262.3	257.9	0.00470	27.660	8.8	9.62
17	244.7	253.5	249.1	0.00472	26.640	8.8	9.67
18	235.8	244.7	240.25	0.00475	25.623	8.9	9.72
19	226.9	235.8	231.35	0.00477	24.609	8.9	9.77
20	218	226.9	222.45	0.00479	23.604	8.9	9.82
21	209	218	213.5	0.00481	22.600	9	9.86
22	200	209	204.5	0.00483	21.598	9	9.90
23	977.1	1001.6	989.35	0.01320	36.414	24.5	27.03
24	952.6	977.1	964.85	0.01332	35.374	24.5	27.28
25	927.6	952.6	940.1	0.01344	34.337	25	27.52
26	902.6	927.6	915.1	0.01355	33.303	25	27.75
27	877.6	902.6	890.1	0.01366	32.280	25	27.97
28	852.6	877.6	865.1	0.01376	31.269	25	28.18
29	827.1	852.6	839.85	0.01386	30.259	25.5	28.38
30	801.6	827.1	814.35	0.01395	29.249	25.5	28.57
31	776.1	801.6	788.85	0.01404	28.249	25.5	28.75
32	750.6	776.1	763.35	0.01412	27.259	25.5	28.91
33	724.6	750.6	737.6	0.01420	26.267	26	29.07
34	698.6	724.6	711.6	0.01427	25.275	26	29.22
35	672.1	698.6	685.35	0.01434	24.281	26.5	29.36
36	645.6	672.1	658.85	0.01440	23.285	26.5	29.49
37	619.1	645.6	632.35	0.01446	22.297	26.5	29.62
38	592.6	619.1	605.85	0.01452	21.316	26.5	29.73
39	575.3	592.6	583.95	0.00914	31.705	17.3	18.73
40	557.9	575.3	566.6	0.00921	30.660	17.4	18.87
41	540.4	557.9	549.15	0.00928	29.619	17.5	19.00
42	522.8	540.4	531.6	0.00934	28.583	17.6	19.13
43	505.1	522.8	513.95	0.00940	27.552	17.7	19.24
44	487.3	505.1	496.2	0.00945	26.524	17.8	19.36

Based on the coma-free configuration in which the off-axis angles *α* and *β* are equal to zero, the fixed angle *θ*_λ_ of each grating listed in column 6 of Table 1 can be simply and precisely calculated with respect to the diffraction direction by using Eq. (4).

4 $$\theta_{\uplambda } = \sin^{ - 1} \left( {\frac{{\uplambda _{o} }}{2d}} \right),$$

where the groove width *d* = 1/*g* and *λ*_*o*_ is the center wavelength of the grating specified in Table 1.

In experiment, two neighboring pixels can be used to resolve two wavelengths according to the following maximum wavelength resolution:

5 $$\Delta\uplambda _{\max } = \frac{dw}{f} > \frac{dw\cos \theta }{f},$$

where *d*, *f* and *θ* are, respectively, the groove width, the focal length of the system and the diffraction angle, and *w* is the photon sensor width equaling to 13.5 µm for the CCD array detector used in the experiment. When gratings with groove densities of 3600 lines/mm, 1800 lines/mm and 1200 lines/mm are used, *d* is approximately 278 nm, 556 nm and 833 nm with calculated Δ*λ*_max_ values of approximately 0.005 nm, 0.010 nm and 0.015 nm in the 200–305 nm, 305–593 nm and 593–1000 nm wavelength regions, respectively. Hence, a theoretical resolution greater than 0.005 nm/pixel, 0.010 nm/pixel and 0.015 nm/pixel in the specified subwavelength regions will be obtained for the spectrometer. The experimentally measured data shown in Figs. 3 and 4 are in good agreement with the design prediction. Therefore, according to the theoretical expectations, a high k data density factor characterized for a high spectral performance of the spectrometer can be realized. The k factor is the ratio of the total wavelength range and the spectral range of each pixel of the photodetector array, i.e., k = (total wavelength range)/(pixel resolution). From our measurements, the k factor is 2.1 × 10^4^, 2.9 × 10^4^, and 2.7 × 10^4^ in the wavelength range of 200–305 nm, 305–593 nm and 593–1000 nm, respectively, resulting in a one-dimensional total of approximately 7.7 × 10^4^ wavelength data points measured at once in the entire 200–1000 nm spectral region without any moving element.

To ensure higher diffraction efficiency, UV-enhanced gratings are chosen for the 200 to 397 nm wavelength region, and gratings with blaze wavelengths of 500 nm, 750 nm and 1000 nm are chosen to operate in the 397–699 nm, 699–903 nm and 903–1000 nm wavelength ranges, respectively. As seen in Fig. 5, there is a set of filter F, in which an empty window in the 200–379 nm wavelength range is designed, and three other filters have cut-off wavelengths of 350 nm, 490 nm and 640 nm to eliminate the high order (*m* ≥ 2) of diffracted light effectively in the 379–593 nm, 593–802 nm and 802–1000 nm wavelength ranges, respectively.

As shown in the data listed in columns 7 and 8 of Table 1, there is a certain redundant wavelength region with a pixel number greater than that required for each grating in the design. Therefore, the wavelength ranges between two neighboring gratings can be seamlessly connected through the whole spectral region without error in practical applications.

The computer-aided software and computer-assisted machining were used to design and produce in high-precision for all mechanical and optical parts of the spectrometer, including the integrated grating module Gx with each fixed sub-angle to mount 44 sub-gratings as shown in Fig. 5. To ensure the long-term stability of the system, afterwards, all parts of the spectrometer were fixed with optical glue.

In the conventional off-axis configuration of a C-T spectrometer, the image of the input slit of the spectrometer will be slightly distorted on the focal plane of the 2D array detector, which is a phenomenon called spectral curvature (or the “smile” effect)^37,38^, which will reduce both the spectral resolution and the accuracy of the spectral measurement. By means of using a zero-off-axis configuration for a C–T spectrometer, the error arising from the “smile” aberration has been effectively overcome in the data calibration procedure. Therefore, the concept of the zero-off-axis configuration presented in this work has unique merit that enables it to be used in many other types of spectrometers for a broad range of applications.

## Summary

In summary, a super-high resolution optical spectrometer with zero off-axis angles was experimentally demonstrated. The new spectrometer configuration completely eliminates the root cause of the coma aberration. In terms of using a 2D array CCD with a 2048 × 2048 pixel density and 13.5 × 13.5 μm^2^ pixel size, a single grating-integrated module consisting of 44 sub-gratings in an optimal arrangement was constructed for the spectrometer to identify the unique spectral “fingerprints” of elements with ultrahigh resolution in a broad wavelength region. By measuring the 44-fold spectra in the 200–1000 nm wavelength region, the physical size of the photoelectron detection area was one-dimensionally extended by a factor of 44, reaching about 1216 mm with more than 9 × 10^4^ pixels along the diffraction direction on the focal plane of the array detector. Hence, the design satisfies the seamless connection requirement between two neighboring sub-spectral regions without any missing wavelengths throughout the entire wavelength range. In tests by a dual-element Hg–Ar light source, the system shows the performance evaluated by the highest spectral data density of the k parameter achieved to date, which is about 2.1 × 10^4^, 2.9 × 10^4^, and 2.7 × 10^4^, in the 200–305 nm, 305–593 nm and 593–1000 nm wavelength regions, respectively. Note that k = (total wavelength range)/(pixel resolution), which corresponds to a one-dimensional total of approximately 7.7 × 10^4^ wavelength data points measured at once in the entire 200–1000 nm spectral region without any moving element. The experimentally measured data have verified that the resolution of the spectrometer is greater than 0.005 nm/pixel, 0.010 nm/pixel and 0.015 nm/pixel in the 200–305 nm, 305–593 nm and 593–1000 nm wavelength regions, respectively, which is in good agreement with the theoretically expected values in the design. Therefore, by using the advanced 2D array detectors and multiple-grating modules, the concept of the zero-off-axis configuration proposed in this work has unique merit that enables it to be used in many other types of spectrometers for a broad range of applications in the future.

## Methods

The spectrometer with a coma-free configuration and a compound integrated grating module is schematically illustrated in Fig. 5, in which the system is designed with zero off-axis angles, *α* and *β*. The module of grating Gx consists of 44 sub-gratings which are in parallel arranged in the direction perpendicular to the incident plane of light. By coupling of the fiber to the slit S with a size of 10 µm (width) and 0.6 mm (height), the light signal is input to the system. The incident light will enter along path L1 through a hole centered between gratings 22 and 23, as shown in Table 1, and then be reflected along path L2 by spherical mirror M1 having a focal length of 750 mm.

The collimated light enters the grating module Gx with a zero off-axis angle of *α* = 0, referring to the conventional C–T configuration shown in Fig. 1. The first order (*m* = 1) of diffraction, which has higher efficiency for all gratings, was applied. The set of filter F was applied to cut off the high order (*m* ≥ 2) of the diffracted light effectively in each sub-spectral range. The light diffracted by the grating module Gx will travel along path L3 and be reflected by toroidal mirror M2, which has two focal lengths, i.e., one is 750 mm in the direction parallel to the incident plane of light, and the other is 915 mm in the direction perpendicular to the incident plane of light. The diffracted light was reflected by mirror M2 along path L4 with a zero off-axis angle of *β* = 0 at the 2D focal plane of the CCD detector (PIXIS-2048-BUVcamera) (https://www.princetoninstruments.com/products/pixis-family), which has dimensions of 2048 × 2048 pixels with a pixel size of 13.5 × 13.5 µm^2^ and a SiO_2_ transparent window to work in the 200–1000 nm spectral range with higher sensitivity in the 200–375 nm range. Thus, the integrated spectra of 44 gratings will be imaged with a size ≤ 27.6 mm at the 750 mm focal position in the directions perpendicular to the incident plane of light and be filled entirely to the focal plane of the CCD detector. By measuring the 44-fold spectra in the 200–1000 nm wavelength region, the physical size of the photoelectron detection area is one-dimensionally extended by a factor of 44, reaching about 1216 mm with greater than 9 × 10^4^ pixels along the diffraction direction at the focal plane of the array detector.

As shown in Fig. 5b, the camera with the 2D BSI-CCD array detector was placed on the top of the grating module with a small off-axis tilt angle of approximately 6° in the direction perpendicular to the diffraction plane without the coma effect. Thus, every sub-spectral region, each with dimensions of approximately 46 × 2048 pixels, has a light-detecting area of about 0.62 mm × 27.6 mm at the 2D focal plane of the detector in the directions perpendicular and parallel to the incident plane of light, respectively.

Given the arrangement of 44 gratings with the parameters listed in Table 1, gratings 11–22 with *g* = 3600 lines/mm and dispersion greater than 0.005 nm/pixel work in the 200–305 nm spectral range, gratings 1–10 and 39–44 with *g* = 1800 lines/mm and dispersion greater than 0.010 nm/pixel work in the 305–593 nm spectral range, and gratings 23–38 with *g* = 1200 lines/mm and dispersion greater than 0.015 nm/pixel work in the 593–1000 nm spectral range.
